# Decontamination of dental implant surface in peri-implantitis treatment: A literature review

**DOI:** 10.4317/medoral.19420

**Published:** 2013-08-29

**Authors:** Ana Mellado-Valero, Pedro Buitrago-Vera, María F. Solá-Ruiz, Juan C. Ferrer-García

**Affiliations:** 1 Prosthodontics and Occlusion Teaching Unit, Faculty of Dentistry, University of Valencia, Spain; 2 Medical-surgical Teaching Unit, Faculty of Dentistry, University of Valencia, Spain; 3 Endocrinology and Nutrition Teaching Unit, Consorcio Hospital General Universitario de Valencia, Spain. Department of Medicine, Faculty of Medicine, University of Valencia, Spain

## Abstract

Etiological treatment of peri-implantitis aims to reduce the bacterial load within the peri-implant pocket and decontaminate the implant surface in order to promote osseointegration. The aim of this literature review was to evaluate the efficacy of different methods of implant surface decontamination. A search was conducted using the PubMed (Medline) database, which identified 36 articles including in vivo and in vitro studies, and reviews of different decontamination systems (chemical, mechanical, laser and photodynamic therapies). There is sufficient consensus that, for the treatment of peri-implant infections, the mechanical removal of biofilm from the implant surface should be supplemented by chemical decontamination with surgical access. However, more long-term research is needed to confirm this and to establish treatment protocols responding to different implant characterics.

** Key words:**Peri-implantitis, treatment, decontamination, implant surface, laser.

## Introduction

Treatments using dental implants to replace missing teeth are effective and predictable and show good long-term success rates (1,2). However, with the ever-growing popularity of implant treatments and the increasing number performed in recent years, the incidence of short-term and long-term complications has increased. One of these complications, which may lead to loss of the implant in the long term, is peri-implantitis (2).

Peri-implantitis has been defined as an inflammatory lesion of the tissues surrounding the implant subjected to functional loading, with a loss of supporting bone.

When affectation is limited to the mucosa and does not involve bone loss, it is known as mucositis (3).

The literature provides widespread evidence of peri-implantitis’s microbial etiology (4), with a microbiota that is very similar to advanced periodontitis, with high levels of spirochetes and non-motile anaerobic Gram-negative bacterium (*Aggregatibacter Actinomycetemcomitans, Porphyromonas gingivalis, Prevotella intermedia, Tannerella forsythia y Treponema denticola*) (5).

According to Teughels et al. (6), in addition to its chemical composition, the implant’s surface roughness has a significant impact on the quantity and quality of the plaque formed. Rough surfaces and those presenting greater surface free energy (as in the case of titanium) tend to accumulate more plaque. Furthermore, initial bacterial adhesion starts in areas of high wettability (a characteristic of titanium) and inside the pits and grooves of the roughened surfaces, where from it is difficult to eliminate.

Longitudinal prospective studies of peri-implant disease are needed to identify the real risk factors for peri-implant disease, but to date few have been published. A systematic review of research published before January 2008 (7) identified much evidence that poor oral hygiene, a history of periodontitis and/or of smoking are indicators of peri-implantitis risk. However, there is no conclusive information relating to the issue of implant surface characteristics as a determining factor for peri-implantitis, and in the few studies that do exist, information is sometimes contradictory. It is not, therefore, surprising that the therapies proposed for treating peri-implantitis are based on the evidence available for the treatment of periodontitis, and are aimed at reducing the bacterial load within peri-implant pockets and decontaminating implant surfaces, and in some cases, attempting afterwards to bring about bone regeneration (4). The therapies proposed include: non-surgical debridement, antimicrobial therapy, surgical access, decontamination of the implant surface, bone regeneration of the defect (when indicated) and supportive therapies.

The objective of the present review was to evaluate the information available in the literature as to the efficacy of different mechanisms for decontaminating implant surfaces in the treatment of peri-implantitis.

## Material and Methods

An Internet search was made using the PubMed (Medline) database. Clinical studies, meta-analyses, clinical guides and reviews published during the last ten years were included. The search strategy used the MeSH browser and the search term *peri-implantitis* and sub-terms *prevention and control and treatment*. The search was then refined using a combination of the terms: *peri-implantitis and decontamination; implant surface decontamination; peri-implantitis and treatment; laser decontamination of implant surface*.

## Results

Of the 135 articles identified in the search only 36 fulfilled the quality criteria as recommended in recent literature (8,9). Studies in which the main topic was regenerative treatment of peri-implant defects were excluded.  Table 1 and  Table 2 show in vivo and in vitro studies identified by the search.

**Table 1:**
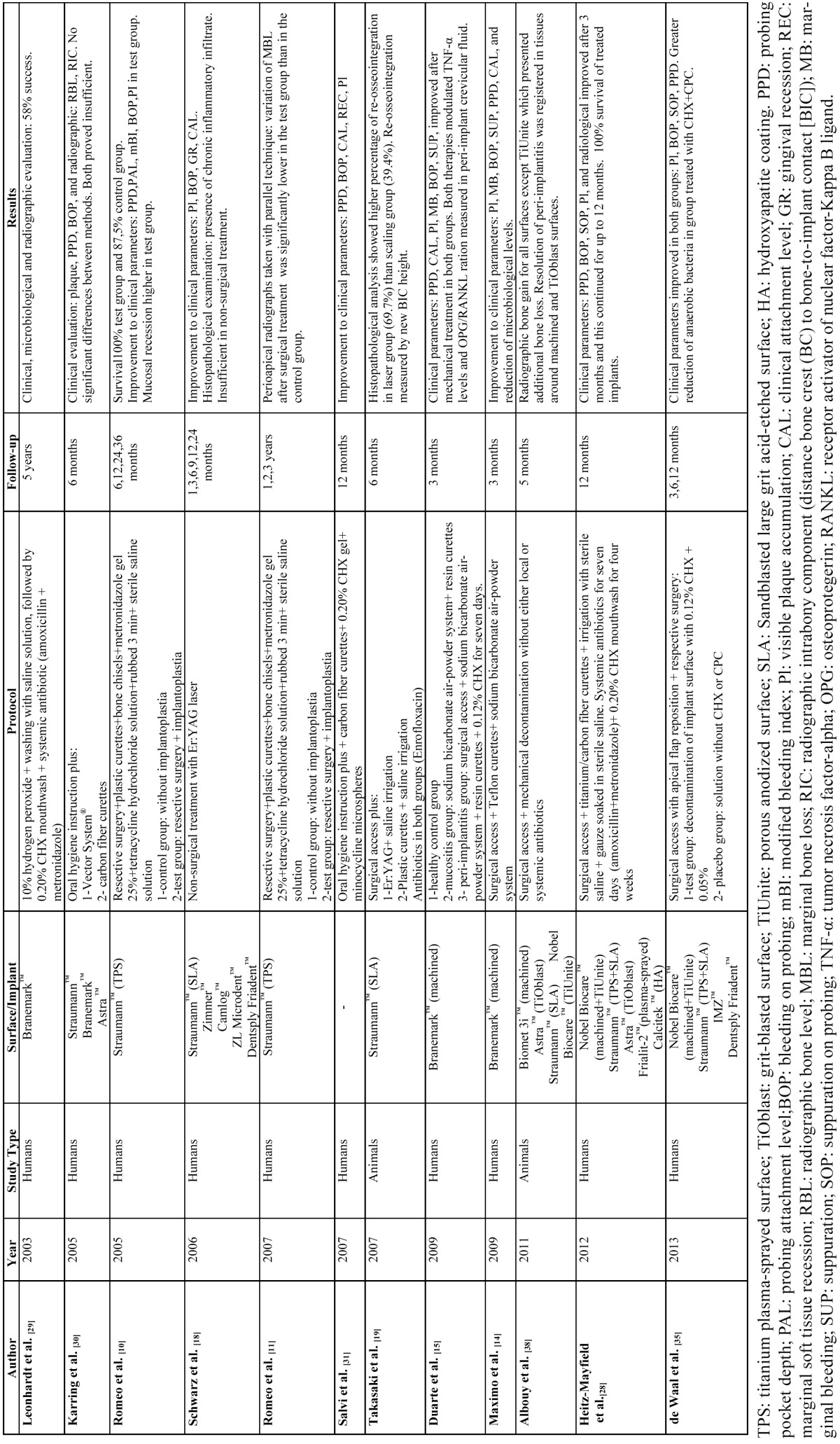
Decontamination of implant surface. In vivo studies (2002-the present) reviewed.

**Table 2:**
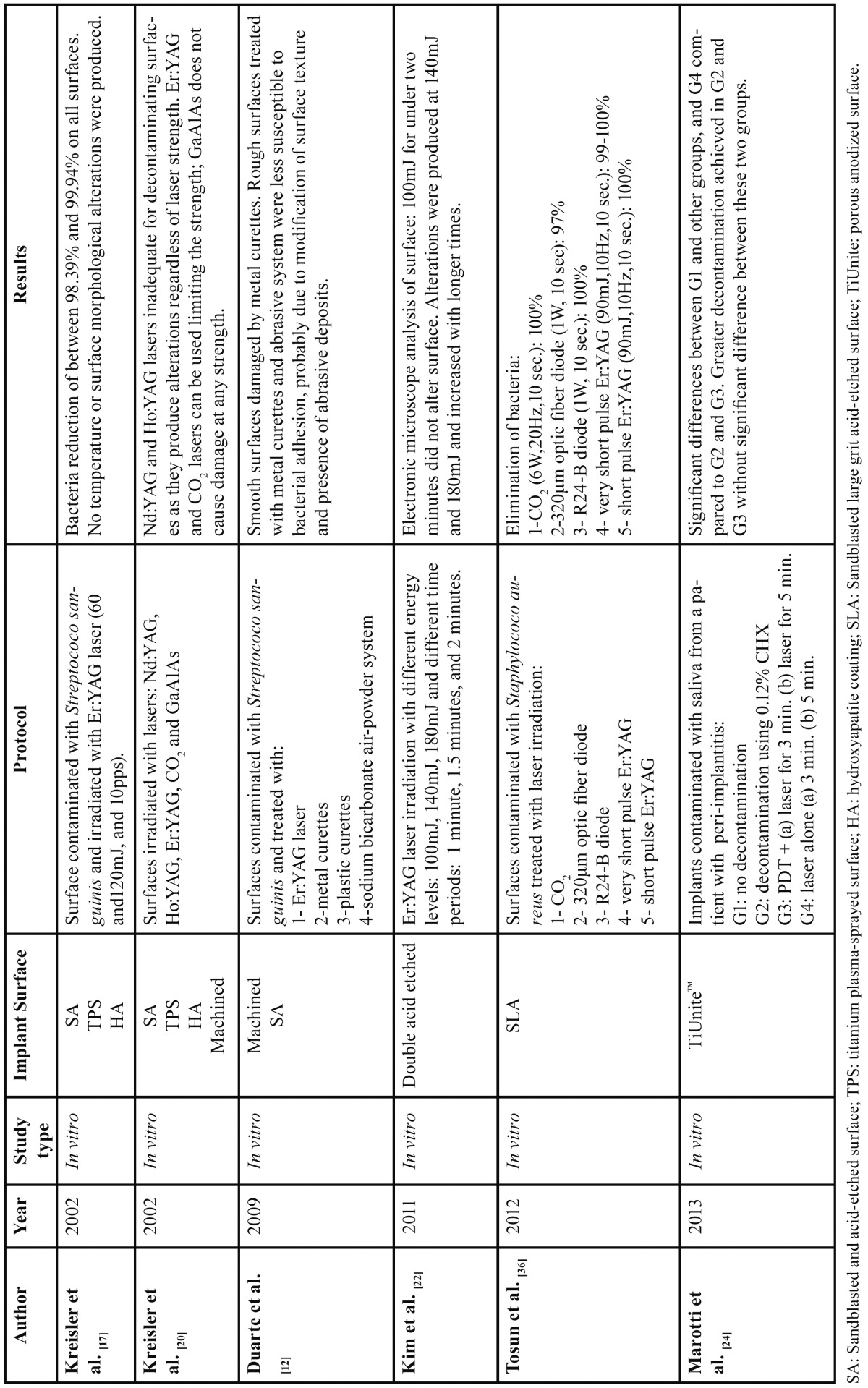
Decontamination of implant surface. In vitro studies (2002-the present) reviewed.

All literature reviewed analyzed the diverse methods of implant surface decontamination which were classified as two main groups: chemical and physical. The latter was sub-divided into mechanical decontamination techiques and laser decontamination techniques. Photodynamic therapy, a technique covered by various authors, falls into either category as it combines light-sensitive chemical agents with lasers used to promote their cytotoxicity. The relevant features of each method are as follows.

-Physical Decontamination Methods.

Mechanical 

The mechanical removal of biofilm from the implant surface is considered a priority for the etiological treatment of peri-implantitis. Its objective is the elimination of toxins from the implant surface in order to produce a surface compatible with health, to promote re-osseointegration. The main difficulty lies in the implant’s surface roughness, which facilitates bacterial adhesion and colonization. One of the techniques proposed for dealing with this is implantoplasty, that is, the mechanical elimination of surface roughness together with the implant thread. This technique allows to optimize the maintenance and facilitates the oral hygiene to the patient when implant threads are exposed. There are few studies that show the clinical and radiological long-term outcomes of implantoplasty. Romeo et al. (10,11) showed 100% of implant survival after 3 years, with improvements in clinical and radiological parameters compared with those without implantoplasty.

Sandblasting systems using different abrasive particles have been used for the surgical treatment of peri-implantitis in animals and humans, without producing adverse effects. The use of this technique on smooth or roughened surfaces makes them less susceptible to bacterial adhesion, possibly because of the modification of the surface texture and because of the presence of abrasive deposits (12), but it is not recommended for the elimination of supramucosal calculus from titanium posts in supportive therapy. The use of metallic curettes has been shown to alter surface roughness favoring bacterial colonization, whereas plastic curettes produce minimal damage or none at all (13).

In peri-implantitis treatment, mechanical debridement of granulation tissue with teflon curettes and abrasive sodium carbonate air-powder, performing full thickness flap elevation, produces clinical (plaque levels, marginal bleeding, bleeding on probing, supuration, probe depth) and microbiological improvements after three months (14,15).

A review realized in 2004 concluded that bicarbonate air-powder abrasion systems and physiological saline obtain the best results for eliminating endotoxins and detritus from all surfaces (16).

-Decontamination Using Laser 

Laser decontamination is based on its thermal effect, which denatures proteins and causes cellular necrosis.

The use of Er:YAG laser has been widely studied in recent years and has been shown to be effective for biofilm removal, having bactericidal effects that do not damage implant surfaces (17). Its use in non-surgical peri-implantitis treatments brings improvements to clinical parameters. However, histopathological observation reveals permanent chronic inflammatory infiltrate (18). But when it is used for degranulation and debridement of implant surfaces in surgical peri-implantitis treatment, it produces up to 69.7% re-osseointegration, observed histologically (19).

Kreisler et al. (20), using scanning electron microscopy (SEM), analyzed the effects produced by Nd:YAG, Ho:YAG, Er:YAG, CO2 and GaAlAs lasers, on four types of implant surface: machined, sandblasted and acid-etched (SA), titanium plasma sprayed (TPS) and hydroxyapatite coated (HA). The results showed that Nd:YAG and Ho:YAG lasers produce significant damage to the surfaces studied, regardless of the strength at which they are applied, rendering them unsuitable for the decontamination of implant surfaces. CO2 and Er:YAG lasers may be used at limited strengths and GaAlAs laser did not alter the surfaces in any way, even at maximum strength, and so was considered the safest for application to any surface type. It has been observed in in vitro studies that the maximum Er:YAG laser strength that can be applied will vary in relation to the surface being treated. Laser pulses of 300mJ/10Hz produce alterations to SLA surfaces and 500 mJ/10Hz pulses will alter polished surfaces; this differs from continuous wave diode and CO2 laser which no not produce any modification (21). For double acid-etched surfaces, laser strength should be in excess of 100 mJ/10Hz for a duration of under two minutes, which will detoxify the implant surface without producing any surface alterations (22).

In agreement with these findings, a literature review showed that the application of CO2 and diode lasers with different wave lengths were effective for eliminating bacteria without producing alterations to the surfaces treated. Nor were significant rises in temperature detected in the implant body (23).

Photodynamic Therapy (PDT)

Photodynamic therapy is a technique that uses a photosensitizing substance that fixes itself to the bacteria of the biofilm, and when irradiated with laser, cytotoxic singlet oxygen is produced which is able to destroy the bacterial cells. The use of PDT and lasers has generated much interest because of its potential for decontamination of implant surfaces in peri-implantitis treatment. A recent review of in vitro studies, which aimed to analyze the effect of laser on titanium surfaces, has shown that it is possible to carry out photosensitization which is lethal to bacteria but does not damage the implant surface (23). PDT appears to be more efficient for eliminating bacteria from implant surfaces than laser irradiation alone. A comparison between four groups (G1: without decontamination; G2: decontamination using chlorhexidine; G3: PDT= laser + methylene blue dye; G4: laser alone) with the use of GaAlAs laser (660nm, 30mW) there were significant differences between G1 and the other groups, and between Group 4 and Groups G2 and G3, The best results were achieved by G2 and G3, without statistically significant difference between these two groups. (24).

-Chemical Decontamination and Antibiotic Therapy 

Chemical decontamination involves the localized use of anti-microbial solutions such as topical chlorhexidine, tetracycline or minocycline, citric acid, hydrogen peroxide or 35% phosphoric acid gel, in combination with mechanical debridement for elimi-nating hard and soft deposits (25). Comparisons of the decontaminating efficacy of these chemical agents have been made mainly by means of *in vitro* studies on different types of implant surface.

Reviews made by various authors conclude that 40% citric acid with pH 1 for 30-60 seconds has proved the most effective agent for the reduction of bacterial growth on HA surfaces, although clinical application at a more acidic pH could affect the peri-implant tissues and if the time of application is prolonged this can affect the union between the HA and the implant body. Chlorhexidine has been seen to be ineffective on HA surfaces. Machined titanium decontaminates more effectively than other surface types, with topical applications of tetracycline as the antibiotic of choice (16,25,26). However, a review by Claffey et al. of a total of 43 experimental and clinical studies (13 of them performed on human subjects), which evaluated different decontamination protocols using sterile saline solution, chlorhexidine, citric acid and hydrogen peroxide, failed to show that any one method was more effective than the others (27).

The use of 35% phosphoric acid gel for treating peri-implant mucositis would appear to achieve microbial reduction but further studies, both *in vitro* and in humans are needed to determine its efficacy for decontaminating implant surfaces (25).

Most *in vivo* studies use empirical combinations of chemical agents and mechanical procedures with or without systemic antibiotic treatment. Generally, whenever surgical approaches are used, these are supplemented by antibiotic treatment. In one study, surgical debridement of inflammatory tissue by means of titanium or carbon fiber curettes, together with the desinfection of the implant surface by copious irrigation with sterile saline solution and by rubbing the implant surface with gauze soaked in the same solution, was shown to be effective over a twelve-month follow-up, with an implant survival rate of 100%; neither resective surgery nor implantoplasty were performed, and the post-operative protocol included the systemic administration of amoxicillin (500 mg) and metronidazole (400 mg) for seven days, together with 0.20% chlorhexidine mouthwash, twice daily for four weeks (28). A similar protocol with a five-year follow-up, using 10% hydrogen peroxide washed with saline solution and supplemented by 0.20% chlorhexidine mouthwash, together with the administration of systemic antibiotics (amoxicillin and metronidazole), obtained a success rate of 58% for the treatment of implants with machined surfaces (29). In another study that used a surgical approach without systemic antibiotic administration, 45% of implants presented some sign of inflammation after a three-month follow-up (14).

## Discussion

Most published research into the treatment of peri-implantitis and the decontamination of implant surfaces has consisted of *in vitro* studies. Few studies of human subjects have been conducted during the last ten years, and most of these have had short follow-up periods.

Given that the presence of micro-organisms is a key factor for the development of peri-implantitis, etiological treatment must seek to reduce the microbial load, eliminate inflammation of the peri-implant mucosa and decontaminate the implant surface in order to preserve supporting bone and then, if possible, bring about the regeneration of the lost bone.

Despite the fact that mechanical debridement has shown itself to be effective for the reduction of the clinical signs of inflammation (14), the results cannot be considered conclusive. Few clinical studies have evaluated mechanical therapies and normally acted as the control group. Most studies have combined mechanical therapies with local antimicrobial, systemic or regenerative therapies, and have failed to provide clear information as to the effects obtained with each individual therapy.

However, in non-surgical treatment of peri-implantitis, mechanical therapy on its own would appear to be insufficient (30). Used in combination with chlorhexidine, it improves the clinical and microbiological parameters slightly, and the addition of local or systemic administration of antibiotics reduces bleeding on probing and probing depth (31,32). For this reason, non-surgical treatment should limit itself to the treatment of mucositis, as it will not resolve inflammatory lesions in cases of bone loss. For peri-implantitis treatment, surgical access is recommended in order to achieve complete removal of granulation tissue and to obtain access for the decontamination of the implant surface (14). In this way, re-osseointegration can take place, and this has been seen to be more pronounced in cases of roughened implant surfaces than machined ones (33).

For the surgical approach, mechanical and chemical debridement are usually accompanied by systemic antibiotics, obtaining better results than when they are not administered (14,28,29). As concluded by Mombelli and Décaillet in their 2011 literature review, the combination of metronidazole and amoxicillin has the potential to overcome a wide range of pathogens often associated with peri-implant disease (34).

Regarding the use of different chemical agents, a single application protocol cannot be established due to the large number of variables in the research published to date. There is only one double-blinded, randomized clinical study that analyzes the decontaminating effect of chlorhexidine (CHX) in combination with cetylpyridinium chloride (CPC), compared with a placebo. The study group showed greater suppression of anaerobic bacteria than the placebo group in the short term. However, the results were not clinically significant and the microbiological effects in the long term remain unknown (35). According to a review of *in vitro* studies, no single chemical agent showed greater effectiveness than the others (27), although in the case of hydroxiapatite surfaces, citric acid would appear to be the agent of choice, while chlorhexidine is ineffective (16,25,26).

Recent studies have shown the usefulness of lasers for decontaminating titanium implants. The most frequently used are Er:YAG, CO2 and diode due to their haemostatic properties, the selective elimination of calculus and bactericidal effects, which achieve complete or almost complete elimination of bacteria from titanium surfaces, providing they are used within the appropriate parameters for each surface type (36). GaAlAs laser has been shown to be one of the safest as it does not alter implant surfaces, regardless of the strength at which it is applied (20). However, not all studies of the exclusive use of laser techniques have obtained complete surface decontamination (17,24). In a literature review by Subramani in 2012, laser combined with chlorhexidine or saline solution was found to achieve greater percentage of re-osseointegration (25).

A possible alternative approach to implant decontamination, is a combination of conventional treatment with photodynamic therapy (PDT). An application of toluidine blue with soft laser irradiation has been shown to significantly reduce the presence of *Aggregatibacter Actinomycetemcomitans, P gingivalis* and *P intermedia* on different implant surfaces, reduce bleeding on probing and inflammation, but more long-term clinical studies are needed to confirm its effectiveness (24,25).

The real influence of decontamination techniques on the implant surface remains unknown as there have been few studies made using human subjects. However, the scant information available does suggest that smooth implant surfaces are less affected by peri-implantitis than roughened ones (37). Animal studies show a more spontaneous progression of peri-implantitis in implants with anodized surfaces (TiUnite®) than on machined, acid-etched or SLA surfaces (38).

## Conclusions

There are few clinical studies in existence that evaluate the etiological treatment of peri-implantitis in isolation. Most combine this with regenerative techniques of the bone defect caused by the disease, a factor that might obscure the true outcomes of decontamination treatments. Nevertheless, there would appear to be sufficient consensus that, for the treatment of peri-implant infections, the removal of the biofilm from the implant surface should be supplemented by chemical decontamination by means of surgical access. Due to the great heterogeneity of studies, which have used empirical combinations of different decontamination methods, and the variablility of the implant surfaces treated, it is impossible to establish a single protocol for implant decontamination for peri-implatitis treatment. Further long-term clinical studies are needed to confirm the results of the present review and to establish treatment protocols in direct relation to each implant and surface type to be treated.
